# Subfunctionalization influences the expansion of bacterial multidrug antibiotic resistance

**DOI:** 10.1186/s12864-017-4222-4

**Published:** 2017-10-30

**Authors:** Elena Perrin, Marco Fondi, Emanuele Bosi, Alessio Mengoni, Silvia Buroni, Viola Camilla Scoffone, Miguel Valvano, Renato Fani

**Affiliations:** 1 Department of Biology, University of Florence, Via Madonna del Piano 6, 50019 Sesto Fiorentino, FI Italy; 2 Department of Biology and Biotechnology, University of Pavia, Via Ferrata 1, 27100 Pavia, Italy; 3 Wellcome-Wolfson Institute for Experimental Medicine, Queen’s University Belfast, 97 Lisburn Rd, Belfast, BT9 7BL UK

**Keywords:** Subfunctionalization, *Burkholderia*, Antibiotic resistance, RND

## Abstract

**Background:**

Antibiotic resistance is a major problem for human health. Multidrug resistance efflux pumps, especially those of the Resistance-Nodulation-Cell Division (RND) family, are major contributors to high-level antibiotic resistance in Gram-negative bacteria. Most bacterial genomes contain several copies of the different classes of multidrug resistance efflux pumps. Gene duplication and gain of function by the duplicate copies of multidrug resistance efflux pump genes plays a key role in the expansion and diversification of drug-resistance mechanisms.

**Results:**

We used two members of the *Burkholderia* RND superfamily as models to understand how duplication events affect the antibiotic resistance of these strains. First, we analyzed the conservation and distribution of these two RND systems and their regulators across the *Burkholderia* genus. Through genetic manipulations, we identified both the exact substrate range of these transporters and their eventual interchangeability. We also performed a directed evolution experiment, combined with next generation sequencing, to evaluate the role of antibiotics in the activation of the expression of these systems. Together, our results indicate that the first step to diversify the functions of these pumps arises from changes in their regulation (subfunctionalization) instead of functional mutations. Further, these pumps could rewire their regulation to respond to antibiotics, thus maintaining high genomic plasticity.

**Conclusions:**

Studying the regulatory network that controls the expression of the RND pumps will help understand and eventually control the development and expansion of drug resistance.

**Electronic supplementary material:**

The online version of this article (10.1186/s12864-017-4222-4) contains supplementary material, which is available to authorized users.

## Background

Antibiotic resistance is a major challenge for the twenty-first century [1]. A large number of bacteria resistant to multiple classes of antibiotics has emerged worldwide, making treatment difficult [2, 3]. Multidrug antibiotic resistance (MDR) is a highly attractive model to study the evolution of gene function since hypermutation, complex interrelationships between drug resistance and fitness, compensatory evolution, and epistasis affect how resistance evolves and spread [4]. Although different mechanisms influence the emergence of a MDR phenotype, high-level intrinsic MDR, particularly in Gram-negative bacteria, stems from the combined action of efflux pumps and adaptive modifications of the cell envelope [2].

The structural components of MDR efflux pumps (MDR EP), usually chromosomally encoded, are evolutionarily conserved within species [reviewed in [3]]. Indeed, MDR EP are ancient elements in bacterial genomes, suggesting that their functions predate the resistance to antibiotics during the treatment of human infections [5]. Bacterial genomes often contain multiple copies of different MDR EP classes, which have arisen by gene duplications [6, 7]. The importance of gene duplication in driving expansion and diversification of drug-resistance mechanisms and its role as an adaptive response to antibiotic treatment are well-known [8–10]. In general, extra gene copies after duplication events are redundant and free from selection pressure, as they do not add anything to the organism’s capacity to perform the original (duplicated) function [11]. The fate of paralogous copies is strongly debated and no single model can include all the possible alternative scenarios [11]. Duplicated genes rarely exhibit de novo functions (neofunctionalization); more commonly, the functions of the original gene(s) are split into multiple functions among the duplicate genes (subfunctionalization) [12]. The split into subfunctions among the different paralogs may occur when the functions of the original gene(s), which were previously under unified genetic control, acquire their own independent regulation [13].

Among MDR efflux pumps, the Resistance-Nodulation-Cell Division (RND) superfamily is particularly intriguing [14]. Members of this superfamily of proton/drug antiporters located in the inner membrane have several roles including bacterial virulence, quorum sensing, plant–bacteria interactions, and detoxification of metabolic intermediates and toxic compounds, such as heavy metals, solvents, or antimicrobials [15]. The regulation of RNDs depends on global and/or local regulators, resulting in a multilayered control of gene expression in response to specific stimuli. The high efficiency of RNDs in extruding substrates is due to their associations with outer membrane channel (OMP) and periplasmic membrane fusion (MFP) proteins. These tripartite complexes extrude molecules to the extracellular milieu preventing their accumulation in the periplasmic space [16]. The genes encoding the three protein components (RND, OMP, and MFP) are often organized in an operon and several different RND operons may be embedded in the same genome [3, 17]. These efflux pumps have been mostly studied in Gram-negative bacteria, such as *Pseudomonas aeruginosa, Escherichia coli*, and *Acinetobacter baumannii* [3]. Recently, attention has also been focused on RND systems of other pathogenic bacteria, such as *Burkholderia* species [18], and more specifically, the *Burkholderia cepacia* complex (Bcc) [19]. Bcc bacteria have been extensively investigated concerning their genome organization and the presence of several copies of MDR systems, especially the RND superfamily [20, 21].

Although rampant gene duplication played a key role in shaping this gene family, the *tempo and mode* of this process remains unknown. The genome of the model strain *B. cenocepacia* J2315 harbors several genes encoding RND proteins of different families [18, 20–22]. Two RND copies (*RND 2* and *4*) have been the focus of our attention since they provide a model to investigate the evolution of paralogs that are functionally distinct but with a high degree of sequence similarity. Previously, we have shown that the RND 2 coding gene is present only in some Bcc species, and a phylogenetic analysis revealed that the amino acid sequences of RND 2 and RND 4 proteins cluster together [20]. Despite this high degree of sequence similarity, only the RND 4 protein plays a key role in the antibiotic resistance of *B. cenocepacia* J2315, since a deletion of the entire RND 4 operon reduces the intrinsic MDR antibiotic resistance of the mutant strain [23, 24]. Further, RND 4 appeared to be particularly important for antibiotic resistance of planktonic bacteria, but less relevant for resistance in biofilm bacteria [25]. RND 4 also contributes to resistance to the biocide chlorhexidine [26] and the 2-thiopyridine anti-tubercular derivative, which is also effective against *B. cenocepacia* [27]. This pump is also associated with the modulation of some virulence factors (motility, biofilm formation, chemotaxis and *quorum sensing*) [24]. A proteomic analysis comparing the parental strain and the deletion mutant suggests a more general role for RND 4 in the physiology of *B. cenocepacia* cells [28]. Conversely, the *Burkholderia* RND 2 protein is not expressed during growth on LB medium, and its role in antibiotic resistance can only be revealed when overexpressed in *E. coli* [29] .

In this study, we provide computational and experimental evidence supporting an evolutionary model on the functional diversification of these two RND superfamily members in *Burkholderia spp.*, providing a framework to understand how these events could modulate antibiotic resistance.

## Results

### Operon structure, comparative genomics and phylogeny

**Fig. 1:**
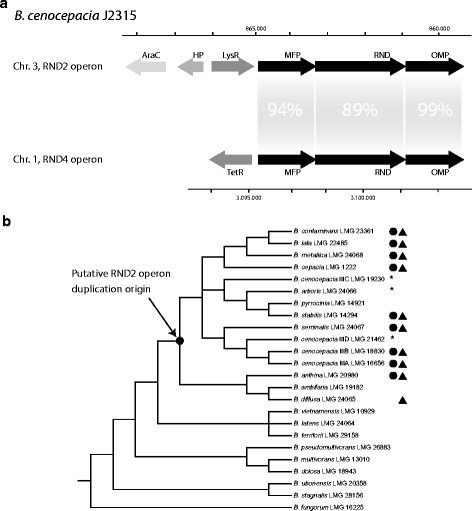
RND 2 and 4 operon structures and hypothetical point of operon duplication. **a**) Schematic representation of RND 2 and 4 operon structures in *B. cenocepacia* J2315 genome. RND 2 operon: MFP coding gene (*BCAS0766 = QU43_RS72495*, 1239 bp), RND coding gene *(BCAS0765 = QU43_RS72490*, 3192 bp), OMP coding gene (*BCAS0764 = QU43_RS72485*, 1503 bp), LysR family transcriptional regulator (*BCAS0767 = QU43_RS72500*, 881 bp) and AraC family transcriptional regulator (*BCAS0768 = QU43_RS72510*, 983 bp). HP: hypothetical protein. RND 4 operon: MFP coding gene (*BCAL2822 = QU43_RS50725*, 1275 bp), RND coding gene (*BCAL2821 = QU43_RS50720*, 3201 bp), OMP coding gene (*BCAL2820 = QU43_RS50715*, 1524 bp), TetR family transcriptional regulator (*BCAL2823 = QU43_RS50730*, 641 bp). The percentages given are the degree of identity between the two operons at the nucleotide level. **b**) Schematic reconstruction of the phylogenetic tree reported in [19]: black triangle = RND 2 operon found in at least one strain belonging to the associated species; black circle = presence of AraC and LysR regulators coding genes associated with the operon; * = no genome belonging to this species is available

The RND 2 operon is located on *B. cenocepacia* J2315 chromosome 3. It consists of three genes organized in the following order: *BCAS0766* (*QU43_RS72495*, 1239 bp), *BCAS0765* (*QU43_RS72490*, 3192 bp), and *BCAS0764* (*QU43_RS72485*, 1503 bp), encoding the MFP, RND permease, and OMP proteins, respectively. A LysR family transcriptional regulator (*BCAS0767*; *QU43_RS72500*, 881 bp) is located upstream of *BCAS0766* and oriented in the same direction (Fig. 1a). Another gene, encoding an AraC family transcriptional regulator (*BCAS0768*; *QU43_RS72510*, 983 bp) is present upstream of the LysR regulator coding gene, but oriented in the opposite direction (Fig. 1a). The RND 4 operon is located on chromosome 1 and spans three genes: *BCAL2822 (QU43_RS50725*, 1275 bp), *BCAL2821* (*QU43_RS50720*, 3201 bp), and *BCAL2820* (*QU43_RS50715*, 1524 bp) encoding the MFP, RND permease, and OMP proteins, respectively. Thus, the RND 2 and RND 4 operons share identical gene organization. A gene encoding a TetR family transcriptional regulator (*BCAL2823*; *QU43_RS50730*, 641 bp), found upstream of *BCAL2822*, is oriented in the opposite direction (Fig. 1a). The degree of identity/similarity between the two operons is 92%, 87%, 95% of identity at the nucleotide level, with 90%, 85%, 96% identity and 93%, 91%, 96% similarity at the amino acid level when comparing the MFP, RND and OMP genes and proteins, respectively. The degree of DNA and amino acid sequence identity/similarity between the genes/proteins of the two operons (Fig. 1a) and the identical gene order strongly suggests these operons arose from a recent operon duplication event. No major evidence of purifying selection was found, as for almost all the pairwise comparisons among the RND, MFP, and OMP sequences, the dN/dS ratio was lower than 1 (Additional file 1 and Additional file 2).

A comparative genome analysis was performed on 797 *Burkholderia* genomes to evaluate the presence, distribution and conservation of RND 2 and RND 4 operons. RND 2 and 4 coding genes were found in 47 and 689 of the genomes analysed, respectively. Then the presence of the other two genes (*mfp* and *omp*) forming the operons was evaluated: the intact RND 2 operon occurred in 33 genomes, while the intact RND 4 operon was present in 472 (Additional file 3). The lack of the RND genes (or of the entire operons) in some strains could be due to the poor quality of the corresponding assemblies (draft genomes). In these genomes, operon fragments were found in contig boundaries. Therefore, incomplete operons were excluded from the downstream analysis. All genomes possessing the RND 2 operon were from Bcc bacterial species, but not all Bcc species harbor this operon. However, except for a few cases, the RND 2 operon co-occurred with RND 4 (Additional file 3). The phylogenetic tree of Fig. 1b (redrawn from the tree reported in De Smet et al. [19]) indicates the species in which a copy of the RND 2 operon was found. According to the phylogenetic distribution of RND4 and RND2 operons, the putative duplication event involving RND 4 and leading to RND 2 can be mapped inside the Bcc group (Fig. 1b).

The conservation of the genes encoding regulatory proteins associated with the RND 2 and RND 4 operons in *B. cenocepacia* J2315 was also investigated. A gene encoding a TetR family transcriptional regulator was always associated with the RND 4 operons identified (Additional file 3). LysR- and AraC-like transcriptional regulator genes present upstream of the J2315 RND 2 operon were also associated with all the RND 2 operons identified, and in the same order and orientation, except for *B. diffusa* (Fig. 1b) (Additional file 3). The RND 4 promoter regions were highly conserved among different strains and divergent from those of RND 2. The RND 2 promoter regions were also conserved among themselves, with the exception of *B. diffusa* (data not shown). In both cases, conserved motifs putatively recognized by regulatory proteins, were identified (Additional file 4).

### Extra copies of the RND 2 operon can functionally replace the RND 4 operon

To assess the role of the two RND operons in antibiotic resistance and whether the RND 2 operon can functionally substitute RND 4, the *B. cenocepacia* J2315 RND 2 and RND 4 operons and their own promoter regions were cloned and inserted into pSCrhaB2 [30], giving rise to two recombinant plasmids (pEP_RND2_operon and pEP_RND4_operon, respectively) (Table 1). These plasmids were introduced in the parental J2315 strain and in a deletion mutant lacking the RND 4 operon (strain D4, Table 1). The antibiotic resistance profile of these strains was investigated by determining the Minimal Inhibitory Concentration (MIC) values of 14 antibiotics belonging to different classes. As a control, the MICs for a RND 2 operon deletion mutant were determined, and the same results were obtained as for J2315 (data not shown). The D4 strain was more sensitive than the parental J2315 to fluoroquinolones, chloramphenicol, tetracycline, rifampicin and novobiocin (Table 2), as previously reported [23, 24]. The other antibiotics tested are not substrates of RND 4 efflux pumps. As expected, the MIC values of D4(pEP_RND4_operon), in which the RND 4 pumps is restored, were identical to those of J2315 (except for levofloxacin, where the complementation of the RND 4 deletion was only partial). These results confirm that the reduction in MIC values was due to the absence of the RND 4 operon. The D4(pEP_RND2_operon) strain, which expresses a plasmid encoded RND 2 pump, showed an identical MIC profile as that of J2315 for ciprofloxacin and norfloxacin, a partial complementation for levofloxacin, sparfloxacin, tetracycline, rifampicin and novobiocin, and a MIC slightly higher for nalidixic acid and chloramphenicol. These results suggest that the RND 2 operon can complement the RND 4 deletion (although to a lesser extent for some antibiotics) despite not being able to carry out this function in the parental strain. The MIC values of J2315(pEP_RND2_operon) strain are not higher than those of J2315, while those of J2315(pEP_RND4_operon) are in some cases slightly higher, showing that additional copies of the two operons do not significantly increase the MIC values of the parental strain.

**Table 1:** Plasmids and strains used in this work

Strain or Plasmid	Description				Souce or reference
Plasmids	Vector	Insert cloned	Insert leight (bp)	Characteristic/genotype	
pGEM-T Easy				Vector for PCR cloning, Amp^r^	Promega
pRK2013				*ori*_colE1_, RK2 derivative, Kan^r^, *mob* ^+^, *tra* ^*+*^	Figurski et al. 1979
pSCrhaB2				*ori*_pBBR1_ *rhaR rhaS P*_*rhaB*_ Tp^r^ *mob* ^+^	Cardona et al. 2005
pEP_RND2_operon	pSCrhaB2	Operon RND_2 (promoter region,BCAS0766–64)	6178	Tp^r^	This work
pEP_RND4_operon	pSCrhaB2	Operon RND_4 (promoter region, BCAL2822–20)	6288	Tp^r^	This work
Strains	Description		Accession number		
* Escherichia coli*					
DH5α	F^−^ Φ80d*lacZ*Δ*MI5* Δ(*lacZYA*-*argF*)*U169 endA* I *recA* I *hsdRI7*(r_K_^−^m_k_^+^) *sup E44 thi-*I Δ*gyrA96 relA* I				Laboratory stock, Bethesda Research Laboratories 1986
SY327	*araD* Δ(*lac pro*) *argE*(Am) *recA56 nalA* λ pir; Rif^r^ anche pRK2013				Miller et al. 1988
SY327 (pRK2013, pEP_RND2_operon)					This work
SY327 (pRK2013, pEP_RND4_operon)					This work
* Burkholderia cenocepacia*					
J2315	CF clinical isolate				G. Manno, Buroni et al. 2009
D4	J2315 ΔBCAL2820–BCAL2822		SRR3736982		Buroni et al. 2009
J2315 (pEP_RND2_operon)					This work
J2315 (pEP_RND4_operon)					This work
D4 (pEP_RND2_operon)					This work
D4 (pEP_RND4_operon)					This work
D4/C_18	D4 derivative, obtained in the presence of Chloramphenicol		SRR3737008		This work
D4/C_20	D4 derivative, obtained in the presence of Chloramphenicol		SRR3737019		This work

**Table 2:** MIC (μg/ml) of different antibiotics for *B. cenocepacia* J2315, D4, D4(pEP_RND4_operon), D4(pEP_RND2_operon), J2315(pEP_RND4_operon), J2315(pEP_RND2_operon), D4/C18 and D4/C20 strains

*B.cenocepacia* strain	Quinolones/Fluoroquinolones					Aminoglycosides	Ansamycins	β_lactams	Macrolides	Tetracyclines	Drug against mycobacteria	Others		
	CIP	LEV	NOR	SPAR	NAL A	KAN	STREP	AMP	ERY	TET	RIF	CHL	GRAM	NOV
J2315	4	8	16–32	8	16–32	2000	>4000	>4000	500	128	256	64	>512	16
D4	2	2	8	4	8	2000	4000	>4000	500	64	64–128	16	>512	<1
D4 (pEP_RND4_operon)	2–4	4	16	8	32	2000	4000	>4000	500	128	256	64	>512	16
D4 (pEP_RND2_operon)	4	4	16	4	64	2000	2000	>4000	500	64–128	128–256	128	>512	8
J2315 (pEP_RND4_operon)	8	8	32	8	32	2000	4000	>4000	500	128	256	128	>512	32
J2315 (pEP_RND2_operon)	4	8	16	8	32	2000	4000	>4000	500	128	256	64	>512	16
D4/C18	8	8	128	4	256	2000	4000	>4000	500	64	64–128	512	>512	64
D4/C20	8	8	32	4	256	2000	4000	>4000	500	64	64–128	512	>512	64

*Abbreviation*: *CIP* ciprofloxacin, *LEV* levofloxacin, *NOR* norfloxacin, *SPAR* sparfloxacin, *NAL A* nalidixic acid, *KAN* kanamycin, *STREP* streptomycin, *AMP* ampicillin, *ERY* erythromycin, *TET* tetracycline, *RIF* rifampicin, *CHL* chloramphenicol, *GRAM* gramidicin, *NOV* novobiocin

Strains information: D4 = ΔRND4 operon; D4 (pEP_RND4_operon) = D4 with the addition of RND4 operon, D4 (pEP_RND2_operon) = D4 with the addition of RND2 operon, J2315 (pEP_RND4_operon) = J2315 with the addition of RND4 operon, J2315 (pEP_RND2_operon) = J2315 with the addition of RND2 operon, D4/C18 and D4/C20 = D4 derived mutant strains, selected on CAF

### Native RND 2 is very poorly expressed in the D4 strain

**Fig. 2:**
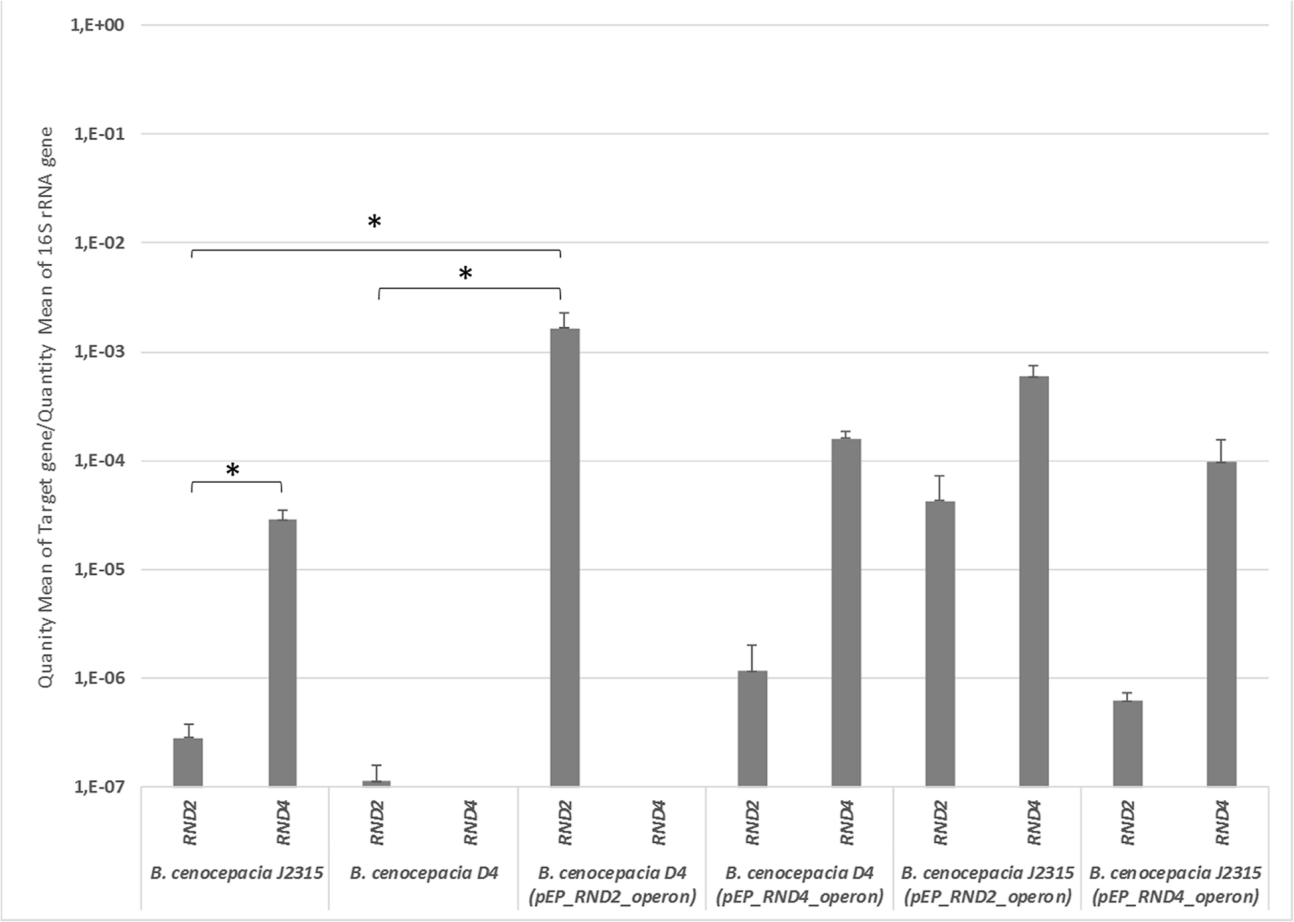
Expression levels of *RND 2- RND 4* genes in the *B. cenocepacia* J2315, D4, D4(pEP_RND2_operon), D4(pEP_RND4_operon), J2315(pEP_RND2_operon), and J2315(pEP_RND4_operon) strains. The quantity mean of RND 2-RND 4 mRNA /quantity mean of the 16S rRNA gene in the presence of 4 μg/ml of nalidixic acid in the *B. cenocepacia* J2315, D4, D4(pEP_RND2_operon), D4(pEP_RND4_operon), J2315(pEP_RND2_operon), and J2315(pEP_RND4_operon) strains are reported. The mean quantity of mRNA was estimated on the basis of the standard curve and for each target gene it was normalized on the mean quantity of 16S rRNA, used as a reference. The differences in genes expression was assessed using a t-test (* = *P* < 0.05)

The previous results indicate that extra copies of the RND 2 operon can functionally replace the RND 4 operon in the D4 deletion mutant by restoring resistance to some antibiotics. This raised the question of why the native RND 2 operon failed to complement the D4 mutation in vivo. To address this question, we investigated the expression of the RND 2 and RND 4 efflux protein genes by qRT-PCR in the presence of nalidixic acid, one of the common substrates of both pumps. In *B. cenocepacia* J2315, the estimated quantity of mRNA of RND 2 was about 100 times lower than that of RND 4 [3·10^−7^ and 3·10^−5^ normalized copies respectively (quantity mean normalized on the quantity mean of the 16S rRNA coding gene)], while no RND 4 operon expression was detected in the D4 mutant (Fig. 2). Further, the copy number of RND 2 mRNA remained low also in the D4 mutant strain (1·10^−7^ normalized copies), suggesting that the loss of RND 4 was not a condition sufficient to increase RND 2 expression. In addition, a strong increase of RND 2 expression was detected in the strain D4(pEP_RND2_operon) (2·10^−5^ normalized copies) (Fig. 2), consistent with the finding that this strain re-acquired resistance to some of the antibiotics tested. The extra-copies of the RND 2 operon in J2315 increased the expression of both RND 4 (6·10^−4^ normalized copies) and RND 2 (4·10^−5^ normalized copies); apparently, this should result in a higher degree of resistance to antibiotics. However, the strong increase of RND 2 expression was masked by the presence of RND 4 efflux pump, which was the main one responsible for the resistance to the antibiotics tested. The addition of extra-copies of RND 4 to J2315 did not significantly increase the expression of RND 4 (1·10^−4^ normalized copies) nor RND 2 (6·10^−7^ normalized copies).

### Spontaneous mutants with high antibiotic resistance can be obtained from the D4 strain through directed evolution experiments

The ability of RND 2 to partially replace the activity of the RND 4 operon through an increase in expression suggests that under selective pressure it should be possible to isolate spontaneous mutants of strain D4 with MIC values similar to those of strain D4(pEP_RND2_operon). Therefore, we set up a directed evolution experiment with the *B. cenocepacia* D4 strain to obtain spontaneous mutants resistant to sublethal concentrations of chloramphenicol (one of the antibiotics that is a substrate of both RND pumps). Two spontaneous mutants, D4/C18 and D4/C20, were obtained after 15 days in the presence of chloramphenicol. These mutants were examined for antimicrobial resistance and the MIC values against different antibiotics for the two mutants were similar (Table 2). Increased resistance against chloramphenicol and novobiocin compared to both D4 and J2315 was observed. For quinolones/fluoroquinolones the behavior was different depending on the antibiotic: nalidixic acid, ciprofloxacin, levofloxacin and norfloxacin had MIC values higher than that of original D4 strain and similar to those of J2315 strain, while sparfloxacin resistance remained that of D4 strain. Therefore, D4/C18 and D4/C20 showed increased multiple antibiotic resistance relative to D4 and not only resistance to the antibiotic used for selection, suggesting the activation of an efflux mechanism.

### An insertion activates RND 2 expression in the D4/C18 and D4/C20 strains

**Fig. 3:**
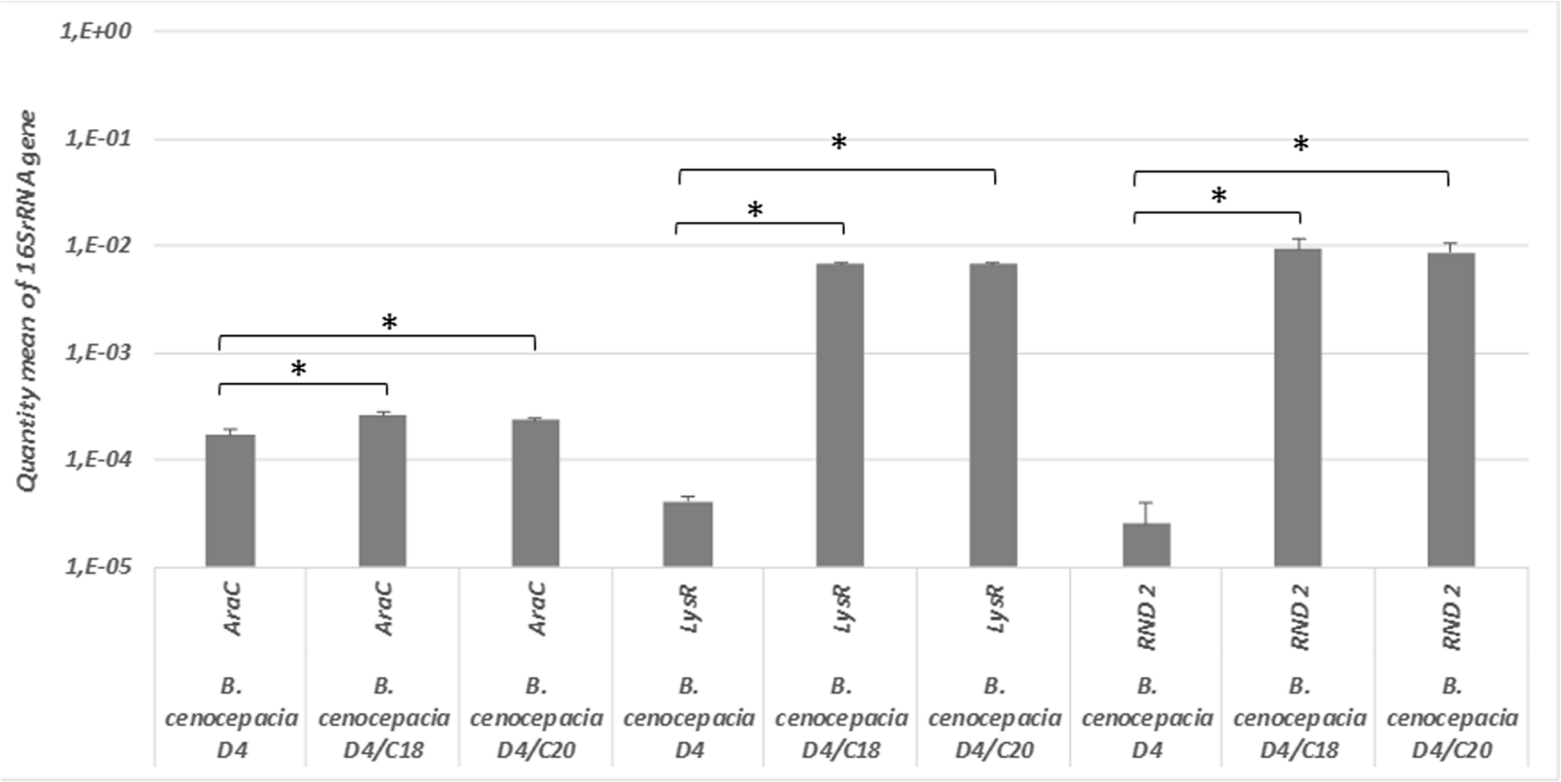
Expression levels of *RND 2, araC* and *lysR* genes in the D4, D4/C18 and D4/C20 strains. The quantity mean of target gene mRNA / quantity mean of 16S rRNA in the *B. cenocepacia* D4, D4/C18 and D4/C20 strains grown in LB medium are reported. The mean quantity of mRNA was estimated on the basis of the standard curve and for each target gene it was normalized on the mean quantity of 16S rRNA, used as a reference. The differences in genes expression was assessed using a t-test (* = P < 0.05)

RAPD fingerprinting analysis confirmed that the D4/C18 and D4/C20 mutants had a profile identical to D4, confirming their clonality (data not shown). We then extracted and sequenced the genomic DNA of strains D4, D4/C18, and D4/C20 to identify the mutation(s) arising under selective pressure (Additional file 5). The genome sequences of the two mutants were compared to those of D4 and J2315 for the presence of SNPs and/or large insertions/deletions. No SNPs were found in the two mutants at the whole genome level, but several deletions and insertions of different size were detected (Table 3). The only mutation common to the D4/C18 and D4/C20 strains, associated with the common phenotype, is a 1300-bp insertion at position 867,051 of chromosome 3, confirmed by PCR and DNA sequencing. This fragment carries predicted transposase and integrase genes both present on chromosome 1 (*BCAL2755* = QU43_RS50390 and BCAL2756 = QU43_RS50395) and chromosome 2 (*BCAM1929* = QU43_RS63980 and BCAM1930 = QU43_RS63985) of J2315. The insertion is located 116 bp upstream of the *araC* family transcriptional regulator gene (*BCAS0768* = QU43_RS72510) and oriented in the same direction, and 482 bp upstream of the *lysR* family transcriptional regulator gene (*BCAS0767* = QU43_RS72500), oriented divergently. These two genes encode regulatory proteins that are associated in the genome with the RND 2 operon. Such genes could be involved in its regulation of expression. To check the possible effects of this insertion on the surrounding genes, the expression levels of both genes and of the RND 2 coding gene were evaluated in D4, D4/C18 and D4/C20 strains (Fig. 3). In both D4/C18 and D4/C20 the expression of the *araC* regulator gene was double that of D4, while the expression of both the *lysR* regulator and *RND 2* genes increased significantly (t-test, *p* < 0.05). We concluded that the increased resistance observed in D4/C18 and D4/C20 was due to increased RND 2 operon expression.

**Table 3:** Mutations identified in *B. cenocepacia* D4/C18 and D4/C20 strains

Strain	Mutations			
*B. cenocepacia* D4/C18	Chromosome	Size (bp)	Position	Genes lost/gained
Deletions	1	2439	From 188,010 to 190,448	BCAL0165, partial BCAL0166
	1	12,975	From 191,455 to 204,429	Partial BCAL0167, BCAL0168–0182
	2	2953	From 2,092,046 to 2,094,998	Partial BCAM1875, BCAM1876, BCAM1876a, Partial BCAM1877
Insertions	1	354	3,135,829	Part of the BCAL2854
	3	320	867,051	Partial BCAL2755, inserted up to BCAS0768
*B. cenocepacia* D4/C20	Chromosome	Size (bp)	Position	Genes lost
Deletions	1	2282	From 188,010 to 190,291	BCAL0165
	1	12,744	From 191,686 to 204,429	Partial BCAL0167, BCAL0168–0182
	1	82	From 1,417,069 to 1,417,150	Partial BCAL1303
	1	10,944	From 1,426,727 to 1,437,670	Partial BCAL1308, BCAL1315–17
	1	197	From 3,554,574 to 3,554,770	No coding region between BCAL3245 and BCAL3246
	3	26,613	From 8888 to 35,500	Partial BCAS0007, BCAS008–0031
	3	79,445	From 493,762 to 573,206	BCAS0421–0503
Insertions	3	886	867,051	Partial BCAS2756 and partial BCAL2755, inserted up to BCAS0768

## Discussion

We investigated how paralog RND operons could have evolved and functionally diversified, and how these events could influence the onset of novel resistance phenotypes. The search for RND 2 and RND 4 operons in 797 *Burkholderia* genomes revealed that the RND 4 operon is highly conserved, being present in almost all the genomes examined. The high conservation of RND 4 is consistent with the critical role of this operon in multiple antibiotic resistance, virulence, and other cellular processes [23–26, 28]. Further, a gene encoding a TetR family transcriptional regulator located upstream of RND 4 is highly conserved, suggesting that regulatory elements controlling the RND 4 operon expression are also common to all *Burkholderia* species.

In contrast, the RND 2 operon is only found in a few Bcc species and likely resulted from a duplication event of the entire RND 4 operon. The conclusion that RND 2 originated by a duplication of RND 4 and not by duplication of other RND operons sharing the very same gene organization [20] is supported by the high degree of sequence identity between RND 2 and RND 4, which is much higher than those existing between RND 2 or RND 4 and the other RND operons. Lastly, the phylogenetic distribution of RND 2 operon allowed us to map the putative duplication event inside the Bcc group (Fig. 1b).

**Fig. 4:**
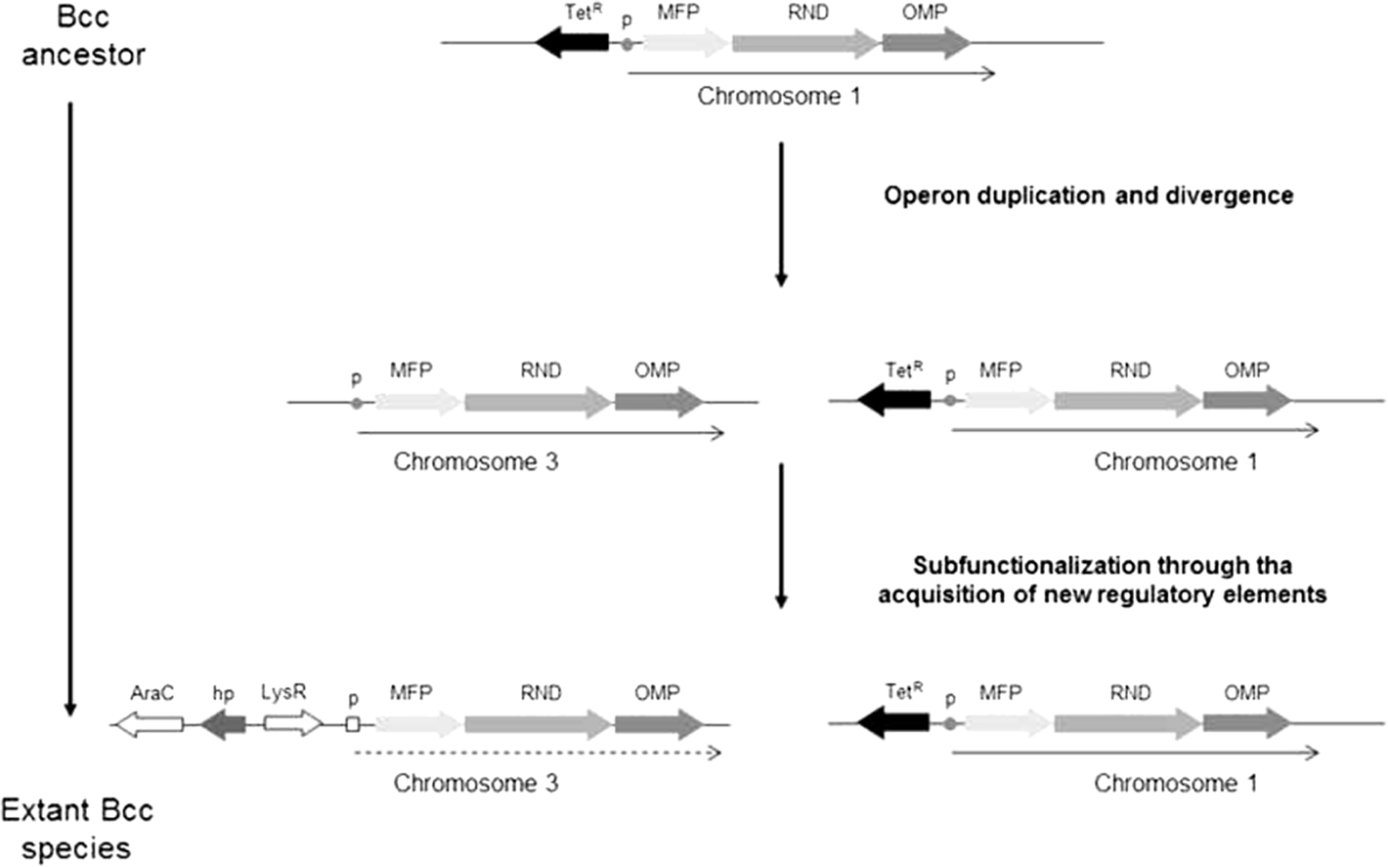
Possible evolutionary pathway leading to the RND 2 and RND 4 operons in Bcc strains

The RND 2 operon has retained most of the ancestral substrate specificity, in that it maintained the ability to extrude the same antimicrobial compounds extruded by RND 4. This hypothesis is supported also by the ability of RND 2 to restore the resistance to some antibiotics in the D4 mutant. However, the recovery of resistance required overexpression of RND 2, that can be obtained either by introducing extra-copies of RND 2 or by mutations altering its regulation. Confirming this scenario, qRT-PCR experiments revealed that RND 2 is expressed at very low level in LB medium even in the presence of a possible inducer, in both *B. cenocepacia* J2315 and the D4 mutant. The need for extra-copies of the RND 2 operon in the D4 mutant or of a mutation to increase the expression of the RND 2 operon suggests that antibiotics are not the *stimulus* that leads to the activation RND 2. According to these hypothesis, different expression “circuits” should control the two operons. From an evolutionary viewpoint, this means that the duplication of the RND 4 operon also involved parallel or subsequent genetic rearrangements resulting in new regulatory elements (e.g. promoters) located upstream of the MFP gene of the RND 2 operon (subfunctionalization) (Fig. 4). This new regulatory control may have involved the appearance of two new genes, encoding AraC-like and LysR-type regulators, respectively (Fig. 1a). These two genes are present in all genomes harboring an RND 2 operon, except for *B. diffusa* (Fig. 1b). This suggests that the molecular rearrangements that led to the localization of these two genes close to the RND2 operon, might have occurred after the duplication event, since *B. diffusa* is phylogenetically located at the base of the cluster in which the duplication occurred (Fig. 1b). The origin of these two genes is still unclear. Searches carried out in all the available prokaryotic genomes did not retrieve any sequence with a degree of identity/similarity sufficiently high to suggest a common origin. Regardless, this regulatory circuit, once acquired, has been maintained, since the two regulatory genes are present and in the same order in all genomes harboring an entire RND 2 operon.

Expression data shed light on the regulation of the RND 2. In the presence of extra copies of RND 2, supplied by the recombinant plasmid pEP_RND2_operon, the expression of RND 2 in J2315 (pEP_RND2_operon) strain is higher than that in the parental strain. A plausible scenario could be that a constitutively expressed repressor controls the RND 2 operon. We can then hypothesize that when extra copies of the RND 2 operon are present, the repressor concentration might be not sufficient to block RND 2 expression. Accordingly, mutations increasing the expression of a regulator gene would have similar effects. This hypothesis was supported by the results from the directed evolution experiments. In fact, increased RND 2 expression was parallel to a strong increase of the associated *lysR* gene expression. Also, the gene encoding the AraC-type protein might play a role in the RND 2 expression, since the expression of this gene increases in both mutants D4/C18 and D4/C20.

In summary, our results show that the Bcc RND 2 operon most likely arose from a duplication of the RND 4 operon, but although RND 2 retains almost the same functions in antibiotics extrusion as RND 4, its expression in *Burkholderia* cells is much lower. Therefore, a different local mechanism regulating the expression of the RND 2 operon suggests that changes in regulatory circuits (rather than functional mutations) may influence the role of different RND paralogs. Interestingly, the fact that the RND 2 operon is encoded by *B. cenocepacia* J2315 chromosome 3 further supports the subfunctionalization hypothesis concerning the evolution of these two efflux systems. Chromosome 3 is a megaplasmid encoding several critical features for pathogenicity and chronic infection, but dispensable for bacterial viability in vitro [31, 32]. This agrees with the notion that the RND 2 operon is not completely diversified from its homolog operon (i.e. RND 4), and consequently, it is not yet essential for *B. cenocepacia* cells. It is plausible that the RND 2 operon may be required only under certain conditions related to chronic infection. Our directed evolution experiment demonstrated that under selective pressure, mutations may arise that rewire the local regulatory circuit of the RND 2 operon, allowing it to respond to the presence of antibiotics. A similar mechanism was identified in *E. coli* cells responding to osmotic stress [33], suggesting that expression rewiring is an evolutionary answer to stress conditions in bacteria.

## Conclusions

Our study contributes to an explanation of how bacterial regulatory networks can evolve to allow partial functional diversification of paralogous genes, thus becoming key players in maintaining a high genomic (and consequently phenotypic) plasticity. Understanding the regulatory network underpinning the expression of the RND multimeric complexes in Bcc can add new information explaining the evolution of drug resistant phenotypes and also potentially new strategies for their control.

## Methods

### Bacterial strains and growth conditions

The bacterial strains and plasmids used in this work are listed in Table 1. Bacteria were grown under aerobic condition at 37 °C in Luria-Bertani (LB) agar or broth. Antibiotic concentration used were 100 μg/ml ampicillin, 50 μg/ml and 50 or 100 μg/ml trimethoprim for *E. coli* and *B. cenocepacia*, respectively. Both antibiotics were purchased from Sigma-Aldrich S.r.l.

### Strains construction

The genome sequence of *B. cenocepacia* J2315 available from GenBank database (GCA_000009485.1) [22], was used for primers design. Due to their high size and GC content, the two RND operons, RND 2 (old locus tags: *BCAS0764–BCAS0766*, new locus tags: *QU43_RS72485, QU43_RS72490*, *QU43_RS72495*) and RND 4 (old locus tags: *BCAL2820–BCAL2822*, new locus tags: *QU43_RS50715*, *QU43_RS50720*, *QU43_RS50725*), were cloned using a two-step strategy. Firstly, a unique restriction site (*Bam*HI for operon RND 2 and *Kpn*I for operon RND 4) was identified in the sequences of the two operons. In this way, the two operons were split into two parts, and two divergent primers (R2_1/F2_2 and R4_1/F4_2 for operon RND 2 and RND 4 respectively, Additional file 6) were designed in correspondence of these two restriction sites. Then, for each operon a forward primer for the amplification of the first part of the operon (including the putative promoter region) and a reverse primer for the amplification of the second part of the operon (including the putative transcription terminator regions) were designed (Additional file 6). These primers contained the restriction sites necessary for the subsequent recovery of the operon (in both cases an *Nde*I and *Hind*III restriction site were introduced in the forward and reverse primers, respectively). The primer pair F2_1/R2_1 was used to amplify a fragment of 3479 bp containing the putative promoter region of operon RND 2, the gene coding for the MFP (*BCAS0766, QU43_RS72495*) and a portion of the RND protein-encoding gene (*BCAS0765, QU43_RS72490*). The primer pair F2_2/R2_2 allowed for the amplification of a 2699 bp fragment containing the second half of the RND coding gene (*BCAS0765, QU43_RS2490*) and the OMP coding gene (*BCAS0764, QU43_RS72485*). For the RND 4 operon, the primer pair F4_1/R4_1 was used to amplify a 1937 bp fragment containing the putative promoter region, the MFP coding gene (*BCAL2822, QU43_RS50725*) and the first half of the RND protein-encoding gene (*BCAL2821, QU43_RS50720*). The primer pair F4_2/R4_2 for the amplification to a 4351 bp fragment containing the second half of the RND coding gene (*BCAL2821, QU43_RS50720*) and the OMP coding gene (*BCAL2820, QU43_RS50715*).

PCR amplification of each region was performed in a 50 μL reaction mixture containing an aliquot of 2 μL of purified DNA [prepared employing the NucleoSpin® Tissue extraction kit (MACHEREY-NAGEL GmbH & Co. KG) following the manufacturer’s instructions], 10 μL of 5X reaction buffer (5X Phusion HF Buffer, Thermo Fisher Scientific), 0.5% DMSO, 0.5 μM of each primer, 200 μM dNTPs and 1 U of *Taq* DNA polymerase (Phusion High-Fidelity DNA Polymerase, Thermo Fisher Scientific). In order to reduce the number of non-specific PCR products, an amplification program with several increasing annealing temperatures was used [34]. For the two primer pairs F2_1/R2_1 and F2_2/R2_2, cycle condition were 98 °C for 3′, followed by 30 cycles of 98 °C for 45″, 60 °C for 45″, 62.5 °C for 45″, 65 °C for 45″, 67.5 °C for 45″, 70 °C for 45″, 72 °C for 2′, and a final extension of 10′ at 72 °C. For the primer pair F4_1/R4_1 cycle condition were 98 °C for 3′, followed by 30 cycles of 98 °C for 45″, 67.5 °C for 45″, 70 °C for 45″, 72 °C for 1′, and a final extension of 10′ at 72 °C. For the primer pair F4_2/R4_2 cycling conditions were 98 °C for 3′, followed by 30 cycles of 98 °C for 45″, 65 °C for 45″, 72 °C for 2′ and 30″, and a final extension of 10′ at 72 °C. Amplification products were loaded on 0.6% agarose gels and amplicons were excised from agarose gel and purified using the MinElute gel extraction kit (Qiagen) according to the manufacturer’s instructions.

Then, 3′ A-overhangs were added to the four purified PCR products, in order to permit their insertion into the appropriate plasmid cloning vector (see below). To this purpose, 0.2 mM dATP, 1X reaction buffer (10X DreamTaq Buffer, Thermo Fisher Scientific) and 2 U of Taq polymerase (DreamTaq DNA Polymerase, Thermo Fisher Scientific) were added to the purified DNA in a final volume of 10 μl, and incubated for 20′ at 72 °C. After that, the four amplicons were cloned into the pGEM®-T Easy Vector (Promega), using the manufacturer’s instructions. The obtained plasmids (Additional file 7) were introduced into *E. coli* DH5α by electroporation [35]. The integrity of the cloned fragments was verified by sequencing of the plasmids at the Genechron laboratory (Ylichron Srl, Italy) using the primers reported in Additional file 6.

The entire RND 2 and RND 4 operons were then assembled in the pSCrhaB2 vector [30]. First the two plasmids pGEM_RND2_second_part and pGEM_RND4_second_part (Additional file 7) were digested with *Bam*HI*-Hind*III and *Kpn*I*-Hind*III, respectively. The obtained fragments, corresponding to the second parts of the two operons, were ligated into the *Bam*HI*-Hind*III and *Kpn*I*-Hind*III digested pSCrhaB2, respectively, to yield the pEP_RND2_second_part and pEP_RND4_second_part plasmids (Additional file 7). Then, the two plasmids pGEM_RND2_first_part and pGEM_RND4_first_part (Additional file 7) were digested with *Nde*I*-Bam*HI and *Nde*I*-Kpn*I*,* respectively, and the obtained fragments were ligated into the *Nde*I*-Bam*HI and *Nde*I*-Kpn*I digested pEP_RND2_second_part and pEP_RND4_second_part, respectively, to obtain the pEP_RND2_operon and pEP_RND4_operon plasmids (Table 1 and Additional file 7). All the restriction enzymes were purchased from Fermentas (Thermo Fisher Scientific), while for the ligation reactions the T4-DNA ligase included in the pGEM®-T Easy Vector kit (Promega) was used.

The two plasmids pEP_RND2_operon and pEP_RND4_operon were introduced in *E. coli* SY327 [36] by electroporation [35] and then mobilized into *B. cenocepacia* J2315 by triparental mating, using the helper plasmid pRK2013 [37, 38]. The presence of plasmids in the final strains was controlled.

### MIC determination

The MIC of different classes of antibiotics were determined: fluoroquinolones (ciprofloxacin, levofloxacin, norfloxacin, sparfloxacin, nalidixic acid), aminoglycosides (kanamycin), ansamycins (streptomycin), β-lactams (ampicillin), macrolides (erythromycin), tetracycline, rifampicin, chloramphenicol, gramidicin and novobiocin. All the antibiotics were purchased from Sigma-Aldrich S.r.l..

The MIC determination protocol was adapted from Ulrich et al. [39]. Briefly, an Over-Night (ON) culture in LB broth (and trimethoprim 50 μg/ml for strains carrying plasmids) of each strain was diluted to a concentration of about 10^6^ CFU/ml. In each well of a 96-well plate, 50 μl of these bacterial suspensions were added to plate wells containing LB medium with the appropriate concentration of antibiotics (and trimethoprim at a final concentration of 50 μg/ml for strains carrying plasmids) to obtain a final bacterial concentration of about 5 × 10^4^ CFU/well. Plates were incubated for 48 h at 37 °C statically and MICs determined both visually and based on the OD_600_ using a Tecan Infinite M200 plate reader (Tecan, San Jose, CA). All MICs were determined in triplicate, and the MIC was defined as the lowest concentration of antibiotics that prevented any detectable growth.

### RNA extraction and reverse transcription

*B. cenocepacia* strains J2315, D4, D4(pEP_RND2_operon), D4(pEP_RND4_operon), J2315(pEP_RND2_operon), and J2315(pEP_RND4_operon) were grown in LB broth in the presence of 4 μg/ml of nalidixic acid (half the MIC of the D4 strain), and trimethoprim 50 μg/ml for strains carrying plasmids, until an OD_600_ ~ 0.5–0.6. 500 μl of each culture were treated with the RNA protect bacteria reagent (Qiagen) and total RNA was extracted using the RNeasy Mini Kit (Qiagen), using the manufacturer’s instruction. DNA was removed from the sample using the RNase-free DNase set (Qiagen).

Extracted RNA was reverse-transcribed using the Superscript II Reverse Transcriptase (Invitrogen) and Random primers (Invitrogen) following the manufacturer’s instruction.

*B. cenocepacia* D4, D4/C18 and D4/C20 strains were grown in LB broth until an OD_600_ ~ 0.5–0.6 and RNA was extracted and reverse-transcribed from 500 μl of each of these cultures following the same procedure.

### Quantitative real-time PCR (qRT-PCR)

qRT-PCR reactions were performed in 10 μl reaction mixture containing an aliquot of 1 μl of cDNA, 5 μl of Maxima Syber Green/Rox qPCR Master Mix (2X) (ThermoScientific) and 1 μg/ml of each primer. For *B. cenocepacia* J2315, D4, D4(pEP_RND2_operon), D4(pEP_RND4_operon), J2315(pEP_RND2_operon), and J2315(pEP_RND4_operon), each cDNA, amplification was carried out using both the primer pairs RND_2_for/RND_2_rev and RND_4_for/RND_ 4_rev (Additional file 6), while 16S RNA encoding gene was used as reference. For the *B. cenocepacia* D4, D4/C18 and D4/C20 strains amplifications were performed using the three primer pairs RND_2_for/RND_2_rev, BCAS0767_for/BCAS0767_rev and BCAS0768_for/BCAS0768_rev (Additional file 6). The expression of each gene in the mutant strain was compared with the expression of the same gene in the D4 strain; 16S RNA encoding gene was used as reference.

Each sample was spotted in triplicate and known amounts of DNA of the *B. cenocepacia* J2315 strain (1–0.1-0.01-0.001 ng) were added to obtain a standard curve for each primer pairs. The reactions were performed on a QuantStudio™ 7 Flex Real-Time PCR System (Applied Biosystems by Life Technologies). Cycling conditions were: hold stage [50 °C for 2′ and 95 °C for 10′], PCR stage [40 cycles of: 95 °C for 30″, 57 °C or 60 °C for 30″ (57 °C for the primer pairs RND_2_for/RND_2_rev and RND_4_for/RND_ 4_rev, 60 °C for other pairs), 72 °C for 15″], melt curve stage [95 °C for 15″ and 60 °C for 1′].

### Directed evolution experiment and mutants fingerprinting

*B. cenocepacia* D4 strain was grown ON (16 h) in LB broth with shaking. 100 μl of this culture (~ 10^8^–10^9^ cells) and of a 10^−1^ dilution were plated on LB agar containing different concentration of chloramphenicol (16, 32, 64, 128 μg/ml). All plates were checked every day, for 15 days, and all of the colonies that grew, were further selected on plates with 128 μg/ml chloramphenicol. In this way 2 spontaneous resistant mutants selected on chloramphenicol (named D4/C18 and D4/C20, Table 1) were obtained.

A RAPD fingerprinting of *B. cenocepacia* D4 strain and of the obtained spontaneous mutants was performed as reported in Mocali et al. [40] using the primers 1253 [41] and AP5 [42].

### DNA extraction and genome sequencing

Genomic DNA of *B. cenocepacia* D4, D4/C18 and D4/C20 was extracted using the CTAB protocol previously described in Perrin et al. [43]. Whole genome shot-gun sequencing was performed by the Institute of Applied Genomics and IGA Technology Services S.r.l. (University of Udine, Italy) using an Illumina (Solexa) HiSeq2500.

The genome sequences determined are available in GenBank, accession numbers: *B. cenocepacia* D4 (SRR3736982), *B. cenocepacia* D4/C18 (SRR3737008), *B. cenocepacia* D4/C20 (SRR3737019).

### Mutant validation

Deletions and insertions were confirmed by PCR amplification of the surrounding regions of the hypothetical mutation using the primers reported in Additional file 6. PCR amplifications were performed using the DreamTaq DNA Polymerase (Thermo Fisher Scientific) in a 10 μl reaction mixture containing 1 μl of same purified DNA, 1X reaction buffer, 0.5 μM of each primer, 200 μM dNTPs and 2 U of *Taq* polymerase. Cycling condition were: 95 °C for 3′, followed by 30 cycles of 95 °C for 10″, 60 °C for 30″, 72 °C for xx” (depending on the size), and a final extension of 10′ at 72 °C.

Amplification products were purified as reported above and sequenced at the Genechron laboratory (Ylichron S.r.l., Italy).

### Blast search of RND 2 and 4 operons

All the genomic sequences of *Burkholderia* representatives available from GenBank (up to October 25th, 2016) were downloaded using in-house bash scripts: 797 sequences were obtained. When not present in GenBank, genome annotation was obtained by applying the PROKKA [44] annotation software to the genome sequence.

The amino acid sequences of RND 4 (gi: 206,561,158) and RND 2 (gi: 197,295,595) from *B. cenocepacia* J2315 were used as queries for a blastp search in all the accessions obtained. For each paralog, only best hits with e-values <= 1e^−15^ were considered. In this way, RND 2 and 4 coding genes were found in 47 and 689 of the genomes analyzed, respectively. Neighboring coding DNA sequences (CDSs) were then compared with the amino acid sequences of OMP and MFP from *B. cenocepacia* J2315 (using blastp, considering only the best hits with e-value <= 1e^−15^) to identify the corresponding homologs in the target genome. The entire RND 2 operon was found in 33 genomes, while the entire RND 4 operon in 472 (Additional file 3). The homologs of AraC (gi: 197,295,598), TetR (gi: 206,561,160) and LysR (gi: 197,295,597) were found in a similar way.

### Sequencing reads analysis and variants calling

Reads quality was assessed using FastQC toolkit (http://www.bioinformatics.babraham.ac.uk/projects/fastqc/). Reads trimming was performed using the dynamic trimming approach implemented in SolexQA [45], using a Phred score of 20 as the base call quality threshold. Trimmed reads were then mapped on the reference genome using BWA with default parameters. Only those SNPs that (i) were not present in the WT strain, (ii) were supported by, at least, five reads, and (iii) had a support greater than 50% were considered for further analysis. SNPs calling was performed using VarScan 2.4.0 [46]. Large variants (insertion and deletion) calling was performed on the sorted BAM file produced by BWA using Pilon 1.16 [47]. All SAM and BAM files manipulation was performed using SAM tools [48].

## Additional files

Additional file 1: Calculation of synonymous/non-synonymous substitution. (ZIP 34380 kb)

Additional file 2: Materials and Methods of the calculation of synonymous/non-synonymous substitution and of the analysis of the promoter regions. (DOC 26 kb)

Additional file 3: Distribution of the RND 2 and 4 operons and of their regulator in *Burkholderia* genomes. (XLS 92 kb)

Additional file 4: Analysis of promoter regions. (ZIP 1251 kb)

Additional file 5: Quality and depth of the sequencing run on *B. cenocepacia* mutants. (XLS 29 kb)

Additional file 6: Primers used in this work. (XLS 40 kb)

Additional file 7: Complete list of all the plasmids and strains used in this work. (XLS 35 kb)

## Abbreviations

Bcc: *Burkholderia cepacia* complex
DMSO: Dimethyl sulfoxide
LB: Luria-Bertani
MDR EP: Multidrug resistance efflux pump
MDR: Multidrug antibiotic resistance
MFP: Membrane fusion protein
MIC: Minimal Inhibitory Concentration
NGS: Next generation sequencing
OMP: Outer membrane channel protein
ON: Over night
qRT-PCR: Quantitative Real Time PCR
RND: Resistance-Nodulation-Cell Division
