# Supplementary figures and images for: Subfunctionalization influences the expansion of bacterial multidrug antibiotic resistance
Source: BMC Genomics. 2017 Oct 30;18:834. doi: 10.1186/s12864-017-4222-4 (PMC5663151; doi:10.1186/s12864-017-4222-4)

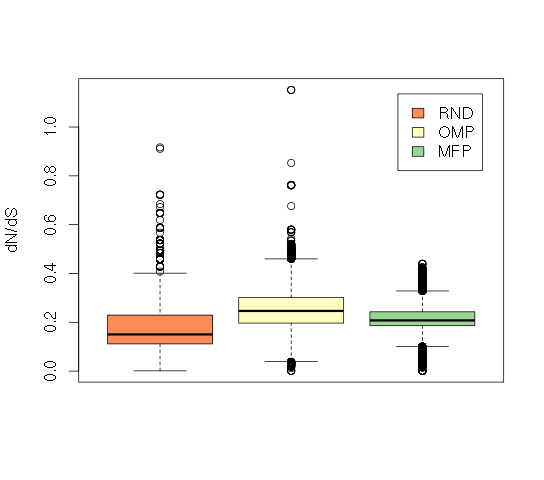

Supplement: Supplementary file 1 — Calculation of synonymous/non-synonymous substitution. (ZIP 34380 kb) [file 12864_2017_4222_MOESM1_ESM.zip › Additional_File_1/Figure_dN_dS.tif]

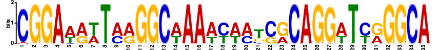

Supplement: Supplementary file 4 — Analysis of promoter regions. (ZIP 1251 kb) [file 12864_2017_4222_MOESM4_ESM.zip › Additional_File_3/Meme_regulators/imgs/motif_1.png]

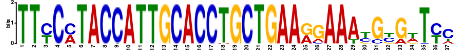

Supplement: Supplementary file 4 — Analysis of promoter regions. (ZIP 1251 kb) [file 12864_2017_4222_MOESM4_ESM.zip › Additional_File_3/Meme_regulators/imgs/motif_2.png]

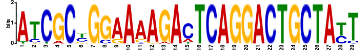

Supplement: Supplementary file 4 — Analysis of promoter regions. (ZIP 1251 kb) [file 12864_2017_4222_MOESM4_ESM.zip › Additional_File_3/Meme_regulators/imgs/motif_3.png]

## Slide: page1
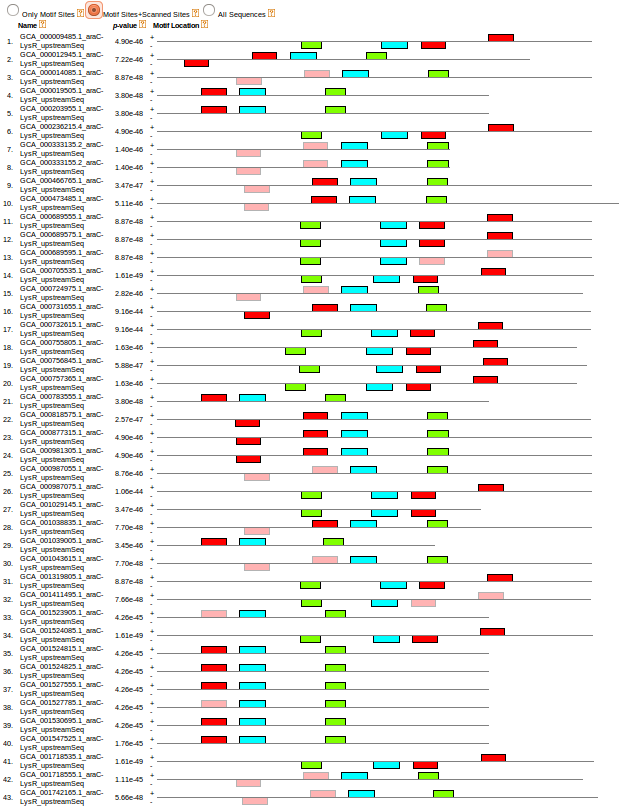

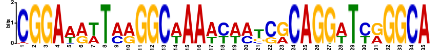

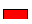

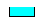

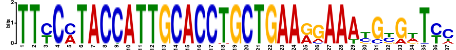

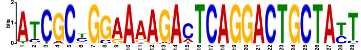

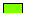


## Slide: page2
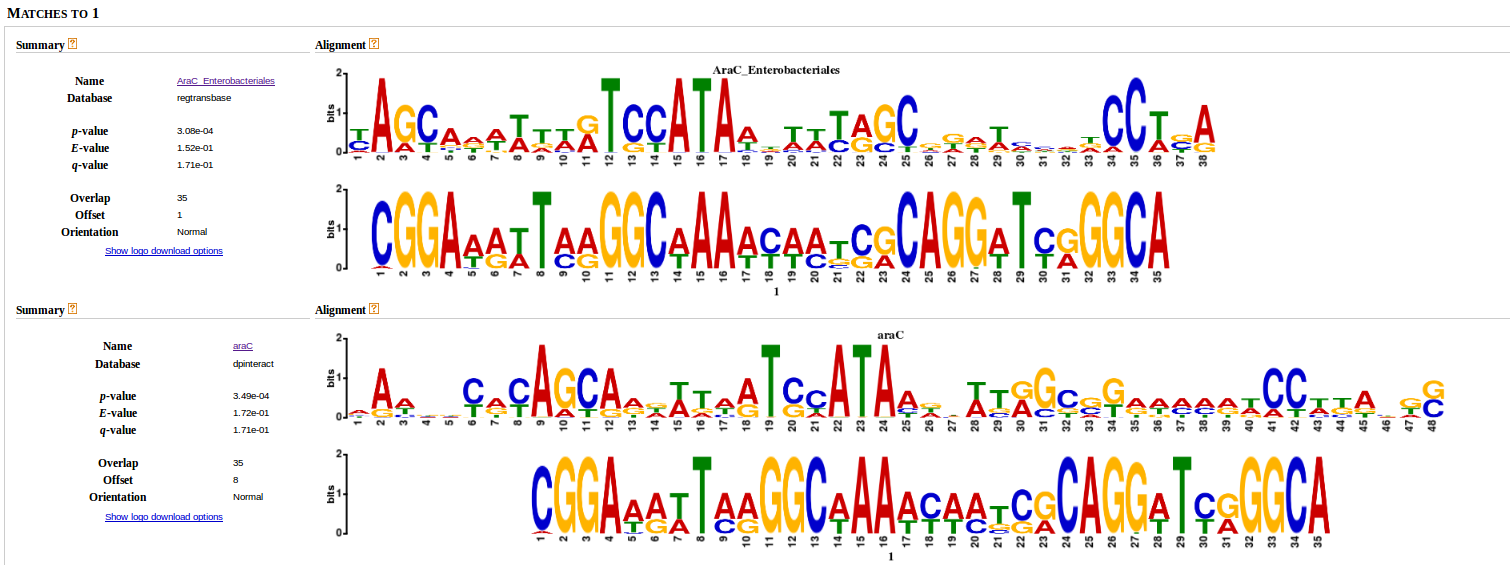

Supplement: Supplementary file 4 — Analysis of promoter regions. (ZIP 1251 kb) [file 12864_2017_4222_MOESM4_ESM.zip › Additional_File_3/Meme_regulators/mix.odp]

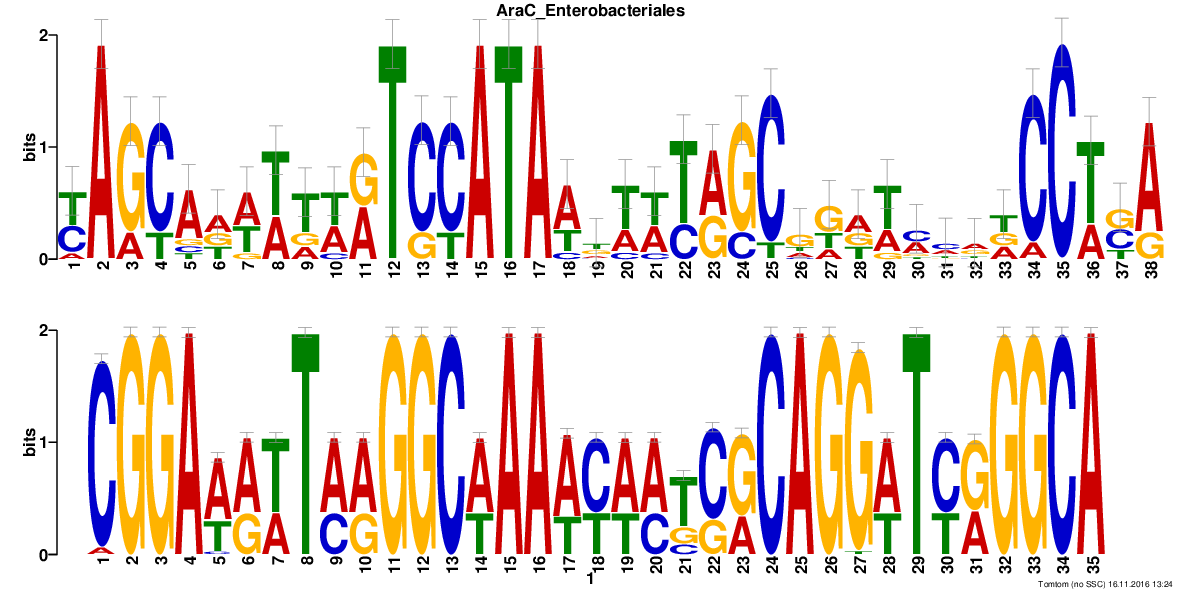

Supplement: Supplementary file 4 — Analysis of promoter regions. (ZIP 1251 kb) [file 12864_2017_4222_MOESM4_ESM.zip › Additional_File_3/Meme_regulators/motif_1_hit_1.png]

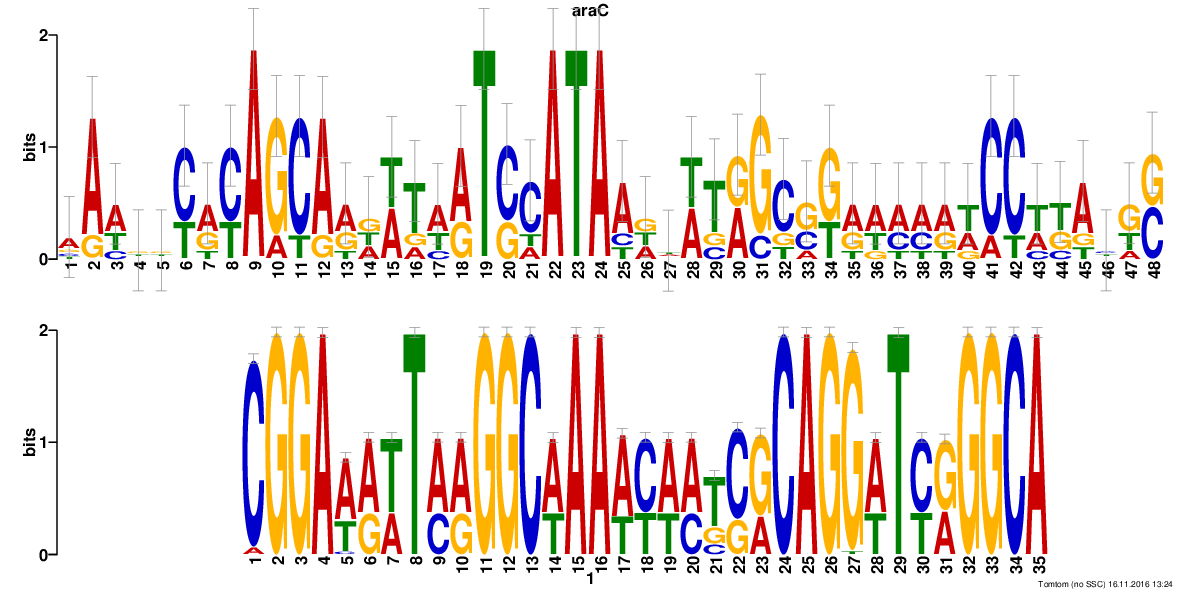

Supplement: Supplementary file 4 — Analysis of promoter regions. (ZIP 1251 kb) [file 12864_2017_4222_MOESM4_ESM.zip › Additional_File_3/Meme_regulators/motif_1_hit_2.png]

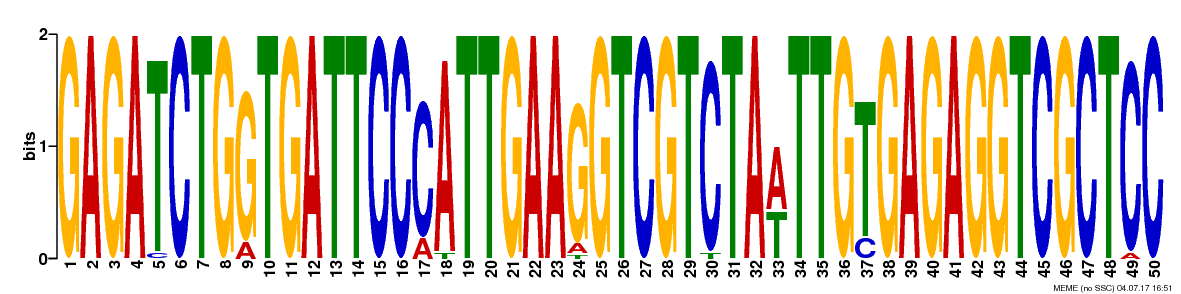

Supplement: Supplementary file 4 — Analysis of promoter regions. (ZIP 1251 kb) [file 12864_2017_4222_MOESM4_ESM.zip › Additional_File_3/Meme_rnd_2/logo1.png]

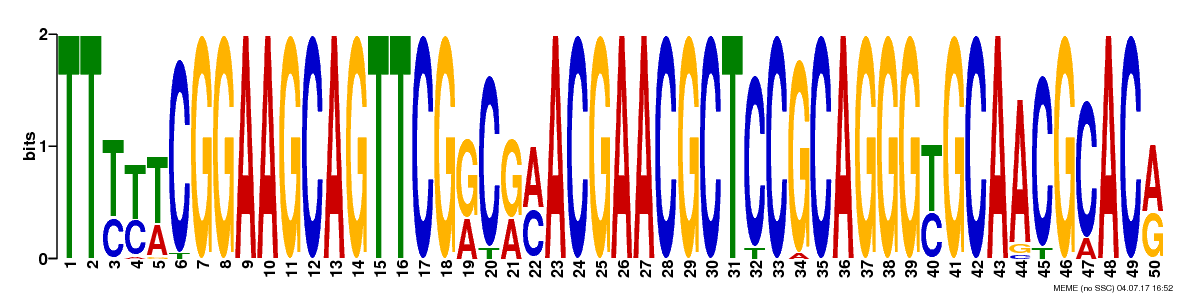

Supplement: Supplementary file 4 — Analysis of promoter regions. (ZIP 1251 kb) [file 12864_2017_4222_MOESM4_ESM.zip › Additional_File_3/Meme_rnd_2/logo2.png]

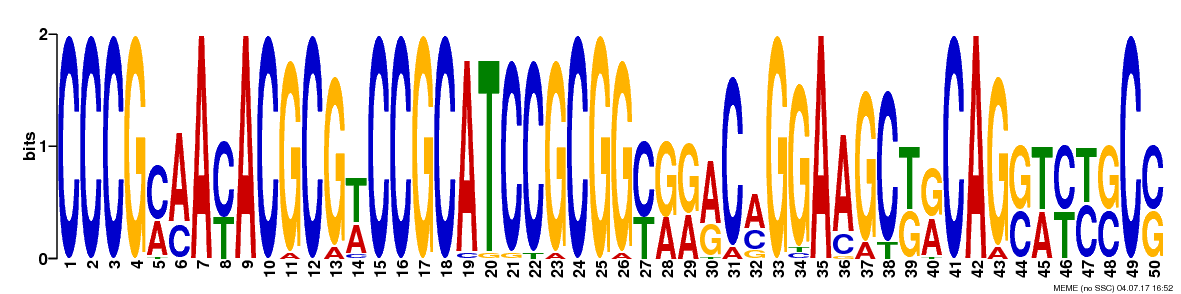

Supplement: Supplementary file 4 — Analysis of promoter regions. (ZIP 1251 kb) [file 12864_2017_4222_MOESM4_ESM.zip › Additional_File_3/Meme_rnd_2/logo3.png]

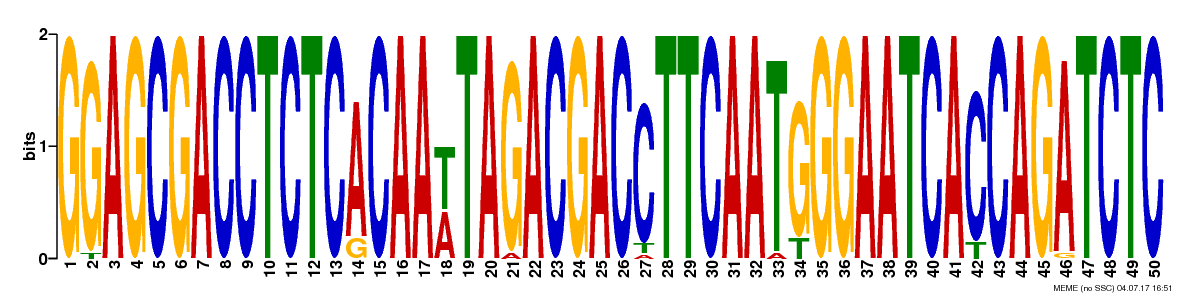

Supplement: Supplementary file 4 — Analysis of promoter regions. (ZIP 1251 kb) [file 12864_2017_4222_MOESM4_ESM.zip › Additional_File_3/Meme_rnd_2/logo_rc1.png]

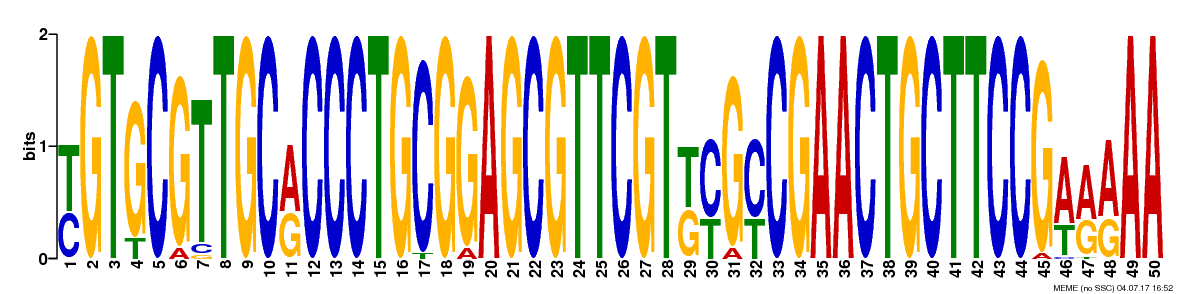

Supplement: Supplementary file 4 — Analysis of promoter regions. (ZIP 1251 kb) [file 12864_2017_4222_MOESM4_ESM.zip › Additional_File_3/Meme_rnd_2/logo_rc2.png]

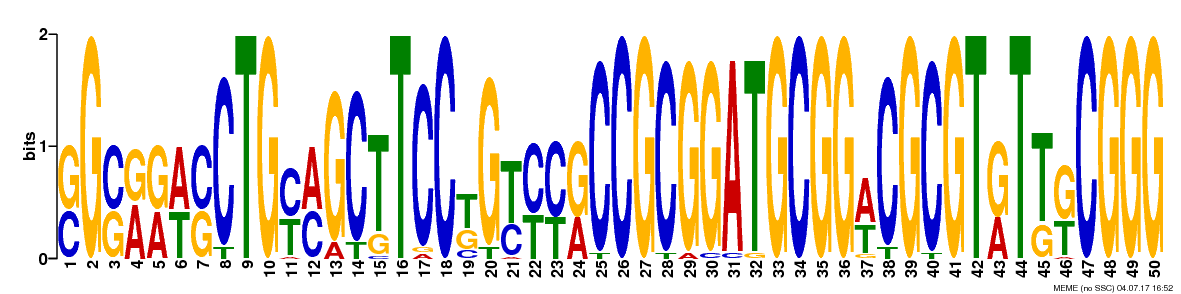

Supplement: Supplementary file 4 — Analysis of promoter regions. (ZIP 1251 kb) [file 12864_2017_4222_MOESM4_ESM.zip › Additional_File_3/Meme_rnd_2/logo_rc3.png]

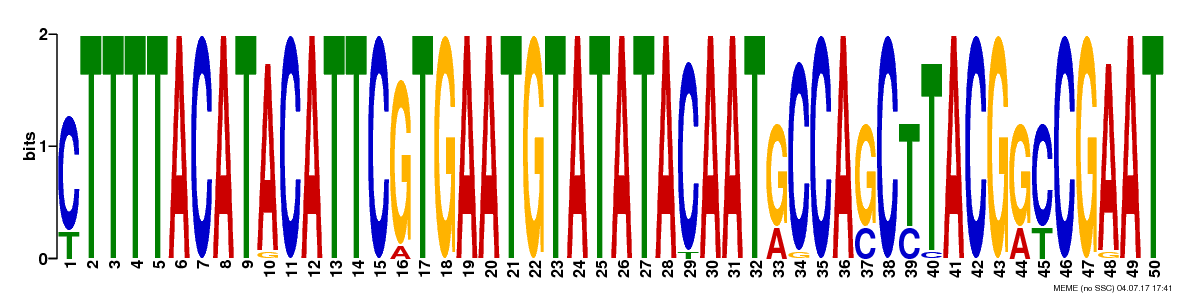

Supplement: Supplementary file 4 — Analysis of promoter regions. (ZIP 1251 kb) [file 12864_2017_4222_MOESM4_ESM.zip › Additional_File_3/Meme_rnd_4/logo1.png]

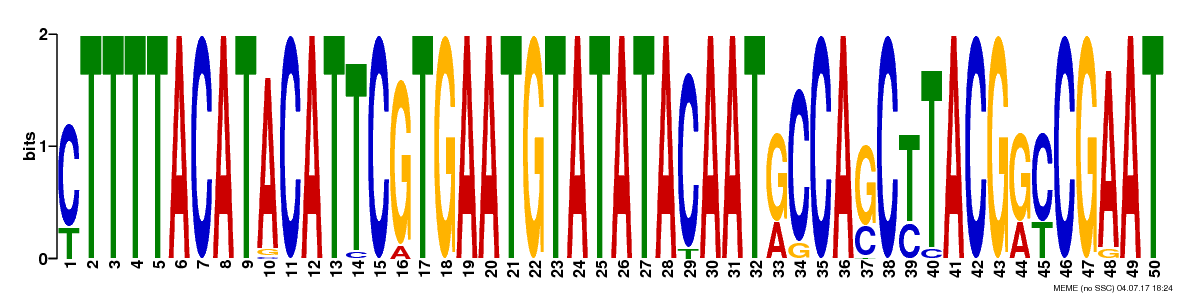

Supplement: Supplementary file 4 — Analysis of promoter regions. (ZIP 1251 kb) [file 12864_2017_4222_MOESM4_ESM.zip › Additional_File_3/Meme_rnd_4/logo2.png]

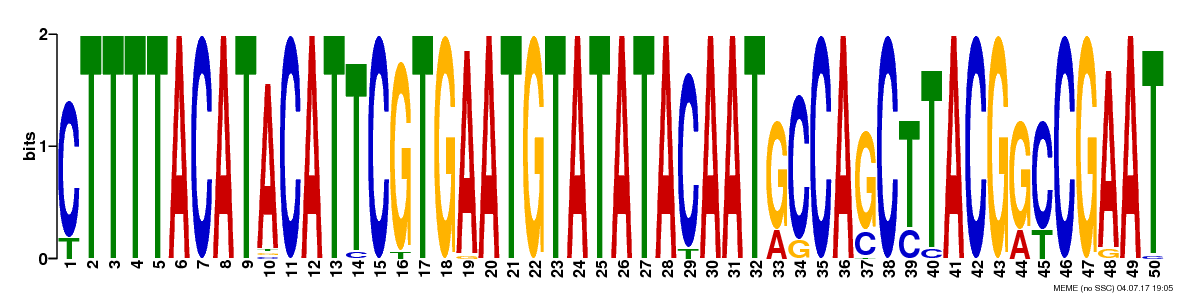

Supplement: Supplementary file 4 — Analysis of promoter regions. (ZIP 1251 kb) [file 12864_2017_4222_MOESM4_ESM.zip › Additional_File_3/Meme_rnd_4/logo3.png]

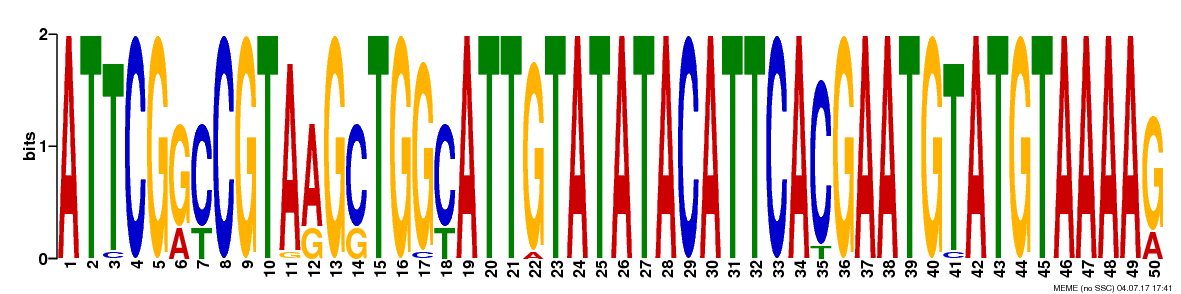

Supplement: Supplementary file 4 — Analysis of promoter regions. (ZIP 1251 kb) [file 12864_2017_4222_MOESM4_ESM.zip › Additional_File_3/Meme_rnd_4/logo_rc1.png]

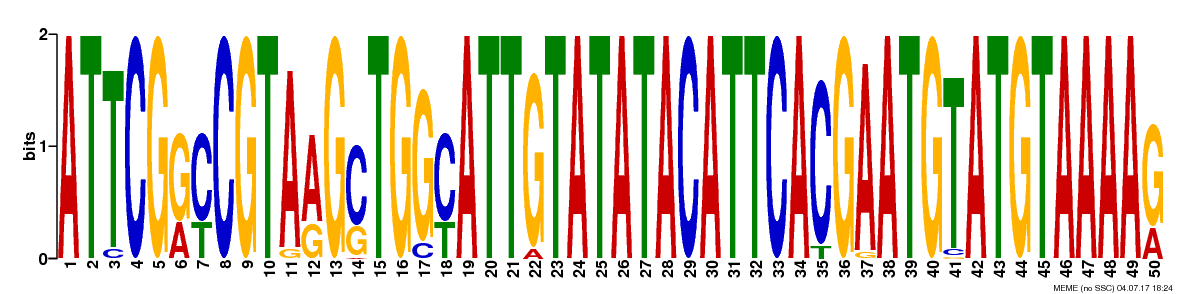

Supplement: Supplementary file 4 — Analysis of promoter regions. (ZIP 1251 kb) [file 12864_2017_4222_MOESM4_ESM.zip › Additional_File_3/Meme_rnd_4/logo_rc2.png]

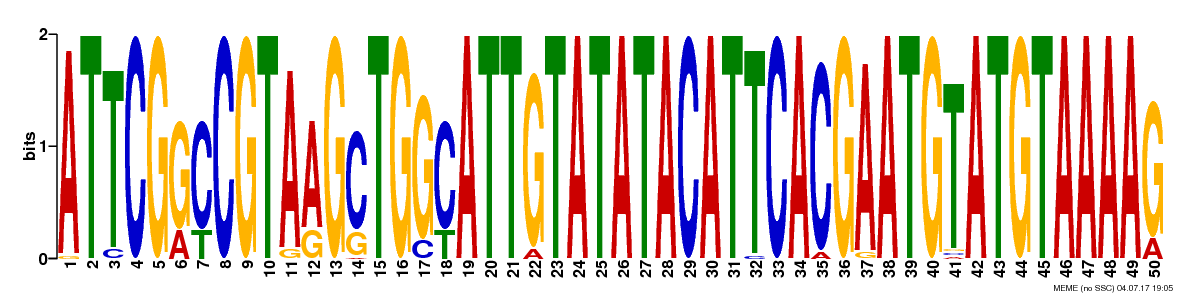

Supplement: Supplementary file 4 — Analysis of promoter regions. (ZIP 1251 kb) [file 12864_2017_4222_MOESM4_ESM.zip › Additional_File_3/Meme_rnd_4/logo_rc3.png]
