# Supplementary material for: Subfunctionalization influences the expansion of bacterial multidrug antibiotic resistance
Source: BMC Genomics. 2017 Oct 30;18:834. doi: 10.1186/s12864-017-4222-4 (PMC5663151; doi:10.1186/s12864-017-4222-4)
Supplement: Supplementary file 2 — Materials and Methods of the calculation of synonymous/non-synonymous substitution and of the analysis of the promoter regions. (DOC 26 kb) [file 12864_2017_4222_MOESM2_ESM.doc]

**Aminoacids substituition rates**

To compute the ratio between synonymous and non-synonymous substitution (Ka/Ks ratio) for RND, OMP and MFP, a codon-based multiple sequence alignment has been obtained for each group of sequences using a command-line version of TranslatorX [1]. The aligned sequences have been analyzed separately with the codeml module of PAML [2], using the following parameters: noisy = 9, verbose = 1, runmode = -2, seqtype = 1, CodonFreq = 0, model = 0, NSsites = 0, icode = 0, fix_kappa = 1, kappa = 1, fix_omega = 0, omega = 0.5.

**Conserved motifs analysis**

A custom python script has been used to extract the upstream sequence of each complete operon, defined as the region comprised between -1 and -300 from the start of the corresponding MFP gene. If one or more genes were present in this range, then the upstream sequence was considered as the region comprised between -1 and the rightmost coordinate found. These sequences were aggregated according to the operon family, and used to identified conserved motifs using the MEME suit (version 4.11.4) [3] with the following command line: meme upstreams.fasta -dna -oc . -nostatus -time 18000 -maxsize 500000 -mod anr -nmotifs 3 -minw 6 -maxw 50 -revcomp.

[1] Abascal F, Zardoya R, Telford MJ: **TranslatorX server: multiple alignment of nucleotide sequences guided by amino acid information*.*** *Nucleic Acids Research* (2010), 38 (suppl_2): W7-W13.

[2] Yang Z: **PAML 4: phylogenetic analysis by maximum likelihood.** *Mol Biol Evol*. (2007), 24(8):1586-91.

[3] Bailey TL, Elkan C: **Fitting a mixture model by expectation maximization to discover motifs in biopolymers.** *Proceedings of the Second International Conference on Intelligent Systems for Molecular Biology*, (1994) pp. 28-36
