# Supplementary material for: Subfunctionalization influences the expansion of bacterial multidrug antibiotic resistance
Source: BMC Genomics. 2017 Oct 30;18:834. doi: 10.1186/s12864-017-4222-4 (PMC5663151; doi:10.1186/s12864-017-4222-4)
Supplement: Supplementary file 4 — Analysis of promoter regions. (ZIP 1251 kb) [file 12864_2017_4222_MOESM4_ESM.zip › Additional_File_3/Meme_rnd_2/tomtom_rnd2.html]

TOMTOM


The database containing the query motif. Refer to the query databases
table for details.

[close ]

The ID of the query motif.

[close ]

The alternate ID of the query motif.

[close ]

A link to more information about the query motif.

[close ]

The motif preview. On supporting browsers this will display as a motif
logo, otherwise the consensus sequence will be displayed.

[close ]

The number of significant matches of the query motif to a motif in the
target database.

[close ]

Links to the first 20 matches of the query motif to a motif in the
target database.

[close ]

The database name.

[close ]

The number of motifs read from the motif database minus the number that
had to be discarded due to conflicting IDs.

[close ]

The number of motifs that had a match with at least one of the query
motifs.

[close ]

The summary gives information about the matched motif. Mouse over each
row to show further help buttons for each specific title.

[close ]

The ID of the matched motif with the optional alternate ID shown in parentheses.

[close ]

The alternative name of the matched motif.

[close ]

The database containing the matched motif.

[close ]

The probability that the match occurred by random chance according to
the null model.

[close ]

The expected number of false positives in the matches up to this
point.

[close ]

The minimum **F**alse **D**iscovery **R**ate required to
include the match.

[close ]

The number of letters that overlaped in the optimal alignment.

[close ]

The offset of the query motif relative to the matched motif in the optimal
alignment.

[close ]

The orientation of the matched motif that gave the optimal alignment.
A value of "normal" means that the matched motif is as it appears in
the database. Otherwise, the matched motif has been reverse complemented.

[close ]

The image shows the alignment of the two motifs. The matched motif is
shown on the top and the query motif is shown on the bottom.

[close ]

By clicking the link "Create custom LOGO ↧" a form to make custom logos
will be displayed. The download button can then be clicked to generate a motif
matching the selected specifications.

[close ]

Two image formats, png and eps, are avaliable. The pixel based portable
network graphic (png) format is commonly used on the Internet and the
Encapsulated PostScript (eps) format is more suitable for publications
that might require scaling.

[close ]

Toggle error bars indicating the confidence of a motif based on the
number of sites used in its creation.

[close ]

Toggle adding pseudocounts for **S**mall **S**ample
**C**orrection.

[close ]

Toggle a full reverse complement of the alignment.

[close ]

Specify the width of the generated logo.

[close ]

Specify the height of the generated logo.

[close ]

|  |  |
| --- | --- |
| Image Type | PNG EPS |
| Error bars | yes no |
| SSC | yes no |
| Flip | yes no |
| Width |  |
| Height |  |

# Tomtom

## Motif Comparison Tool

For further information on how to interpret these results or to get a
copy of the MEME software please access
http://meme.nbcr.net.

If you use TOMTOM in your research, please cite the following paper:  

Shobhit Gupta, JA Stamatoyannopolous, Timothy Bailey and William Stafford Noble,
"Quantifying similarity between motifs",
*Genome Biology*, **8**(2):R24, 2007.
[full text]

Query Motifs  |  Target Databases  |  Matches  |  Settings  |  Program information

## Query Motifs

Next Top

| Database | ID | Alt. ID | Preview | Matches | List |
| --- | --- | --- | --- | --- | --- |
|  |  |  |  |  |  |

## Target Databases

Previous Next Top

| Database | Used | Matched |
| --- | --- | --- |
|  |  |  |

## Matches to

Previous Next Top

| Summary | Alignment |
| --- | --- |
| |  |  | | --- | --- | | Name |  | | Database |  | |  |  | | --- | --- | | *p*-value |  | | *E*-value |  | | *q*-value |  | |  |  | | --- | --- | | Overlap |  | | Offset |  | | Orientation |  |  Show logo download options | ⤒ ↥ ⇘ ↧ ⤓ |

## Settings

Previous Next Top

#### Background

#### Other Settings

|  |  |
| --- | --- |
| Strand Handling | Reverse complements are not possible so motifs are compared as they are provided. Motifs are compared as they are provided. Motifs may be reverse complemented before comparison to find a better match. |
| Distance Measure | Average log-likelihood ratio Euclidian distance Kullback-Leibler divergence Pearson correlation coefficient Sandelin-Wasserman function Bayesian Likelihood 2-Components score (from 1-component Dirichlet prior) Bayesian Likelihood 2-Components score (from 5-component Dirichlet prior) Log likelihood Ratio score (from 1-component Dirichlet prior) Log likelihood Ratio score (from 5-component Dirichlet prior) |
| Match Threshold | Matches must have a E-valueq-value of  or smaller. |

Previous Top

##### TOMTOM version

 (Release date: )

##### Reference

Shobhit Gupta, JA Stamatoyannopolous, Timothy Bailey and William Stafford Noble,
"Quantifying similarity between motifs",
*Genome Biology*, **8**(2):R24, 2007.

##### Command line

  
  
Result calculation took  seconds
